# The Lysosomal Clock: Are We Only as Old as Our Lysosomes?

**DOI:** 10.1111/tra.70056

**Published:** 2026-10-20

**Authors:** Alexis Diaz Vegas, Thierry Galli

**Affiliations:** ^1^ School of Medical Sciences, Faculty of Medicine The University of Sydney Sydney New South Wales Australia; ^2^ Charles Perkins Centre The University of Sydney Sydney New South Wales Australia; ^3^ Université Paris Cité, Institute of Psychiatry and Neuroscience of Paris, INSERM U1266, Membrane Traffic in Healthy & Diseased Brain Paris France; ^4^ GHU Paris Psychiatrie & Neurosciences Paris France

## Abstract

Lysosomes are a major organelle involved in intracellular degradation, membrane traffic and metabolic signaling, but how their biochemical composition changes during normal aging remains poorly defined. Puszynska and colleagues now combine lysosome purification with metabolomics and lipidomics across tissues, species and ages to reveal a conserved signature of lysosomal aging characterized by accumulation of glycerophosphodiesters (GPDs) and cystine and remodeling of bis(monoacylglycero)phosphate (BMP). Strikingly, these changes are largely obscured in whole‐tissue analyses and resemble metabolic abnormalities associated with lysosomal storage disorders, including CLN3 disease and cystinosis. The signature develops progressively with age, can predict chronological age, and is attenuated by caloric restriction in peripheral tissues but not in the brain. Within the brain, age‐dependent metabolite accumulation is particularly prominent in microglia and is also detected in neurons, linking lysosomal aging to cell populations central to neurodegenerative disease. These findings define an organelle‐level metabolic clock, suggesting that one might be only as young as one's lysosomes. Establishing whether GPDs, cystine and BMP remodeling merely mark lysosomal age or actively contribute to declining lysosomal degradation and membrane traffic may uncover a metabolic connection between physiological aging, lysosomal storage disorders and vulnerability to late‐onset neurodegeneration.

## Graphical abstract

The lysosomal clock of aging. Aging is associated with lysosomal accumulation of glycerophosphodiesters (GPDs) and cystine, alongside remodeling of bis(monoacylglycero)phosphate (BMP). This conserved metabolic signature predicts chronological age, is attenuated by caloric restriction in peripheral tissues, and is prominent in brain microglia and neurons, suggesting a potential link between physiological aging, lysosomal storage disorders, and neurodegeneration.

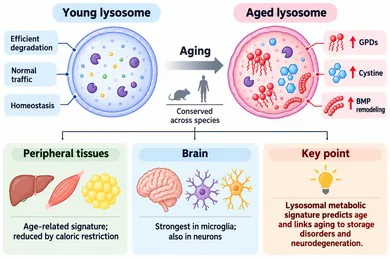

1

Lysosomes sit at the intersection of degradation, membrane traffic and metabolic signaling, yet most descriptions of their decline with age have remained indirect. Whether aging leaves a reproducible biochemical signature that reflects changes in lysosomal cargo degradation and recycling remains unknown. Furthermore, whole‐tissue omics can identify broad age‐associated changes, but signals originating from an organelle that occupies only a small fraction of cellular volume are easily diluted. Puszynska and colleagues now overcome this limitation by profiling purified lysosomes across tissues, species, ages and interventions. Their tour‐de‐force study reveals a surprisingly compact signature of lysosomal aging: the accumulation of glycerophosphodiesters (GPDs) and cystine, accompanied by remodeling of the lysosome‐enriched lipid bis(monoacylglycero)phosphate (BMP) [1].

The authors first use LysoTag mice, in which an HA‐tagged lysosomal membrane protein enables rapid immunopurification, to establish the “Lysosomal MeTabula Muris Senis” across 12 tissues. Although aging reshapes the lysosomal metabolome in a tissue‐dependent manner, brain, heart, skeletal muscle and white adipose tissue share strong increases in cystine and several GPDs, including glycerophosphocholine, glycerophosphoethanolamine and glycerophosphoinositol. These changes are barely detectable in matched whole‐tissue extracts, elegantly illustrating how organelle purification uncovers biology hidden by bulk measurements. Importantly, selective lysosomal rupture supports a luminal localization of the metabolites rather than nonspecific association with the purified fraction.

A major technical advance is the complementary tag‐free method based on immunocapture of endogenous LAMTOR1/p18. This approach reproduces the signature in wild‐type mice, works with frozen material and extends the analysis to two rat strains and archived rhesus macaque muscle. The convergence across genetic backgrounds and species argues against an artifact of LysoTag expression or husbandry. Lipidomics further identifies a compositional increase in BMP/PG species in aged brain, heart and muscle lysosomes without a comparable increase in the major membrane phospholipids. Because BMP supports lipid sorting and degradation within late endosomes and lysosomes, its remodeling may reflect an adaptive response to accumulating lipid catabolic products, altered intraluminal membrane dynamics or both. This could therefore influence membrane organization/fluidity, lipid handling and cargo trafficking, potentially creating a feedback loop between lysosomal membrane remodeling and impaired degradation.

The molecular parallel with lysosomal storage disorders is particularly provocative. CLN3 loss in Batten disease impairs lysosomal clearance of GPDs, whereas defective cystinosin traps cystine in cystinosis [2]. Aging therefore appears to reproduce, slowly and incompletely, metabolic states caused acutely by monogenic defects. The analogy should not be overextended: the study identifies shared storage products, not a generalized lysosomal storage disorder and the upstream age‐dependent lesion remains unknown. Reduced export, impaired hydrolysis, altered lipid delivery, changes in luminal pH, membrane trafficking or a combination of these factors could all converge on the same metabolite pattern. Moreover, whether GPDs, cystine and BMP species are toxic drivers, compensatory products or readouts of declining flux remains unresolved. This distinction matters because lowering a marker will be beneficial only if it interrupts, rather than merely conceals, the pathogenic process.

The longitudinal experiments strengthen the case that this is an aging program rather than an end‐stage consequence. GPDs and cystine rise from early adult life and track chronological age in both sexes. An elastic‐net model using six lysosome‐enriched metabolites predicts age with high accuracy in brain and skeletal muscle, establishing a compelling organelle‐based metabolic clock. Whether this clock also captures biological age, however, remains unknown. Determining whether deviations from the expected lysosomal aging trajectory predict tissue dysfunction or healthspan will be an important next step. Caloric restriction attenuates the signature in heart and muscle, showing that it is physiologically modifiable, but notably fails to do so in the brain. One possibility is that nutrient prioritization and brain‐sparing mechanisms contribute to this tissue‐specific response, although this remains speculative. It also cautions against treating systemic longevity interventions as uniformly effective.

Cell type‐resolved LysoTag experiments provide the clearest route toward neurological relevance. Aging‐associated storage is strongest in microglia and is also evident in neurons, whereas astrocytes and oligodendrocytes are largely spared. These are precisely the cell populations in which lysosomal failure can couple inflammation, defective aggregate clearance and neuronal vulnerability. This cell‐type specificity raises an important question: is lysosomal aging determined by intrinsic differences in lysosomal maintenance, or by the type and amount of cargo that individual cells must process? The result resonates with the view of the aging lysosome as a catalyst for late‐onset neurodegeneration [3]. In Alzheimer's disease, defective autolysosomal acidification and proteolysis can promote intraneuronal amyloid accumulation [4]; in Parkinson's disease, lysosomal genes and pathways involving GBA1, ATP13A2 and other endolysosomal regulators are central determinants of risk and pathology [5]. The newly defined metabolite signature could therefore mark a pre‐disease state that lowers the threshold for proteotoxic, mitochondrial or inflammatory insults.

The next step is mechanistic. Where does the defect lie? (e.g., in cargo delivery to lysosomes, intralysosomal degradation, metabolite export or lysosomal adaptation). Is lysosomal aging predetermined by our genes, or can the environment reshape it? Does experimentally increasing or clearing individual GPDs or cystine alter lysosomal acidification, hydrolase activity, membrane fusion, autophagic flux, lysosomal exocytosis or extracellular‐vesicle release, which is mediated by endolysosomes? Can the signature be detected in human brain, cerebrospinal fluid cells or disease‐relevant induced pluripotent stem cell models? Spatial analyses will also be essential because vulnerable neuronal compartments, particularly long axons and synapses, may age differently from the cell body. Combining lysosome‐resolved metabolomics with measurements of membrane traffic and degradative flux should reveal whether the clock records time or helps drive it.

In conclusion, this study turns the vague concept of an “aging lysosome” into a measurable biochemical state. Its greatest importance for brain disease may lie not in providing another age biomarker, but in identifying a potentially actionable metabolic bridge between normal aging, lysosomal storage disorders and adult neurodegeneration. If GPD and cystine accumulation proves causal, restoring lysosomal export or lipid catabolism could become a strategy to increase resilience in neurons and microglia before irreversible pathology emerges. Tell me how old your lysosomes are, and I'll tell you how old you are!

## Funding

The authors have nothing to report.

## Conflicts of Interest

The authors declare no conflicts of interest.

## Data Availability

The data that support the findings of this study are available from the corresponding author upon reasonable request.
